# Supplementary material for: Content-rich biological network constructed by mining PubMed abstracts
Source: BMC Bioinformatics. 2004 Oct 8;5:147. doi: 10.1186/1471-2105-5-147 (PMC528731; doi:10.1186/1471-2105-5-147)
Supplement: Additional File 2 — The original results of the above study (non-essential files are deleted to keep the file size under the limit set by BMC bioinformatics). [file 1471-2105-5-147-S2.bz2 › chilibotAdditionalFile2/dip20/476ID10734104E10561/html/CCL8_CCRL1.html]

 


 **CCL8** and **CCRL1** 
  
Found 16 abstracts in PubMed, retrieved 16.  
 

 What does Google say? 
 PDF only 
| .edu only 

---

**Interactive relationship** (e.g. stimulation, inhibition, etc)

**Neutral relationship**- Monocyte chemotactic protein 2  [ **CCL8** ]  MCP 2  [ **CCL8** ]  uses CCR1  [ **CCRL1** ]  and CCR2B as its functional receptors.  Ref: 9115216 J Biol Chem, 1997
- Thus, although MCP 2  [ **CCL8** ]  can bind to and signal through CCR1  [ **CCRL1** ] , CCR2b, and CCR5, among which both CCR2 and CCR5 are expressed at high levels on activated T cells, it appears to preferably utilize CCR5 on these cells.  Ref: 9790730 Cell Immunol, 1998
- In experiments using cells transfected with C C chemokine receptors, 125I MCP 2  [ **CCL8** ]  bound to human embryonic kidney 293 cells transfected with CCR1  [ **CCRL1** ]  or CCR2B, known to also bind MIP 1alpha RANTES and MCP 1, respectively, but both shared by MCP 3.  Ref: 9115216 J Biol Chem, 1997
- Both CCR1  [ **CCRL1** ]  and CCR2B transfected 293 cells showed significant migration in response to MCP 2  [ **CCL8** ] , in addition to responding to other specific chemokines.  Ref: 9115216 J Biol Chem, 1997
- MCP 2  [ **CCL8** ]  is also a member of the CC chemokine subfamily and has the capacity to interact with at least two receptors including CCR 1  [ **CCRL1** ]  and CCR2B.  Ref: 9468473 J Biol Chem, 1998

**Non-interactive relationship** (e.g. studied together, co-existance, homology, etc.)

- We used radioligand binding and 35S GTPgammaS exchange assays in membranes from a cell line transfected to express CCR1  [ **CCRL1** ]  Ba F3 hCCR1 to characterize a panel of chemokines HCC 1, MIP 1alpha, MIP 1beta, MIP 1delta, MPIF 1, MCP 2  [ **CCL8** ] , MCP 3, and RANTES as CCR1  [ **CCRL1** ]  ligands.  Ref: 12381680 Br J Pharmacol, 2002
- These results clearly demonstrate that MCP 2  [ **CCL8** ] , unlike MCP 1, uses both CCR1  [ **CCRL1** ]  as well as CCR2B as its functional receptors, and this accounts for the unique activities of MCP 2  [ **CCL8** ] .  Ref: 9115216 J Biol Chem, 1997
